# Legal Aid Societies

**Published:** 1911-12-09

**Authors:** W. R. Bisschop

**Affiliations:** Hon. Sec. The Central Legal Aid Society. 3 Paper Buildings, Temple, E.C.


### The Editor's Letter Box.

### LEGAL AID SOCIETIES.

*To the Editor of* The Hospital.

Sir,—My attention has been drawn to an article in your issue of November 18 headed "Legal Aid Societies." Kindly allow me to inform you of the existence of the Central Legal Aid Society, of which I enclose the rules.

The fact that England is behind Scotland in the meting out of justice to the poor is principally due to the unsatisfactory provisions made for proceedings *in formâ pauperis.* Unless the Rules of Court for such proceedings are made more elastic, there will always remain a difficulty in taking up cases on behalf of the poor.—I am, Sir, your obedient servant,

W. R. Bisschop, Hon. Sec.
The Central Legal Aid Society.

3 Paper Buildings, Temple, E.C.

November 30, 1911.

